# Global Phenotypic Characterization of Effects of Fluoroquinolone Resistance Selection on the Metabolic Activities and Drug Susceptibilities of *Clostridium perfringens* Strains

**DOI:** 10.1155/2014/456979

**Published:** 2014-12-21

**Authors:** Miseon Park, Fatemeh Rafii

**Affiliations:** Division of Microbiology, National Center for Toxicological Research, U.S. FDA, Jefferson, AR 72079, USA

## Abstract

Fluoroquinolone resistance affects toxin production of *Clostridium perfringens* strains differently. To investigate the effect of fluoroquinolone resistance selection on global changes in metabolic activities and drug susceptibilities, four *C. perfringens* strains and their norfloxacin-, ciprofloxacin-, and gatifloxacin-resistant mutants were compared in nearly 2000 assays, using phenotype microarray plates. Variations among mutant strains resulting from resistance selection were observed in all aspects of metabolism. Carbon utilization, pH range, osmotic tolerance, and chemical sensitivity of resistant strains were affected differently in the resistant mutants depending on both the bacterial genotype and the fluoroquinolone to which the bacterium was resistant. The susceptibilities to gentamicin and erythromycin of all resistant mutants except one increased, but some resistant strains were less susceptible to amoxicillin, cefoxitin, ceftriaxone, chloramphenicol, and metronidazole than their wild types. Sensitivity to ethidium bromide decreased in some resistant mutants and increased in others. Microarray analysis of two gatifloxacin-resistant mutants showed changes in metabolic activities that were correlated with altered expression of various genes. Both the chemical structures of fluoroquinolones and the genomic makeup of the wild types influenced the changes found in resistant mutants, which may explain some inconsistent reports of the effects of therapeutic use of fluoroquinolones on clinical isolates of bacteria.

## 1. Introduction

*Clostridium perfringens*, in addition to being the second most common cause of bacterial foodborne illness in the United States [1], may cause other illnesses, including nonfoodborne gastrointestinal illness, antibiotic-associated diarrhea, gas gangrene, septicemia, and enteric diseases in animals [2]. As a colonic bacterium,* C. perfringens* may come in contact with antimicrobial agents used for the treatment and prophylaxis of infections, and large concentrations of ciprofloxacin have been detected in fecal samples after administration of this drug [3]. Early fluoroquinolones were not effective against anaerobes [4];* C. perfringens* strains resistant to these drugs were found in clinical isolates as early as 1992 and in food isolates more recently [5, 6]. Newer fluoroquinolones, however, are more effective and are among the drugs recommended for treatment of* C. perfringens* infections [7].

Fluoroquinolones are DNA-damaging agents; they also induce mutations in gyrase and topoisomerase genes. The mutations in gyrase, topoisomerase, and efflux pump may confer fluoroquinolone resistance on bacteria. Fluoroquinolones also trigger the SOS response and induce DNA repair genes. This may alter the expression of genes involved in the regulation of metabolic activities and lead to phenotypic changes in fluoroquinolone-resistant strains [8, 9]. Excessive use of fluoroquinolones in hospitals has been associated with the emergence of highly virulent strains of* C. difficile* [10]. An* in vitro* study showed that exposure of* C. difficile* to fluoroquinolones resulted in increased toxin production in one strain and decreased toxin production in another strain, indicating a strain-dependent response [11].* In vitro* and* in vivo* studies have also shown that exposure to fluoroquinolones alters the susceptibility of bacterial strains to other antimicrobial agents [9, 12, 13]. Isolation of an extended-spectrum *β*-lactamase-resistant* Escherichia coli* sequence type ST131 with a distinctive virulence profile has been associated with fluoroquinolone resistance [12]. Studies of nosocomial infections in hospitalized patients show that use of levofloxacin or ciprofloxacin is associated with the isolation of methicillin-resistant* Staphylococcus aureus* strains [13]. Contradictory results have been published on the effect of fluoroquinolones on survival and virulence in* E. coli* [14–18].

An* in vivo* study has shown that acquisition of a high level of ciprofloxacin, moxifloxacin, or levofloxacin resistance increases the colonization rate of* C. difficile* strain BI17 in hamsters but that only moxifloxacin resistance increases the colonization rate of* C. difficile* strain BI1 [10]. We have shown that gatifloxacin resistance selection in different strains of* C. perfringens* affects production of short-chain fatty acids, reductive and hydrolytic enzymes, and toxin expression in different ways [19–21]. Fluoroquinolone resistance selection also affects bacterial fitness, and we have shown that resistance selection to different fluoroquinolones has various effects on the fitness of different strains of* C. perfringens* [22, 23]. To investigate the effect of resistance selection to fluoroquinolones with different structures on the metabolic activities of resistant mutants, we used Biolog phenotype microarrays, which detect cellular phenotypes by measuring bacterial growth under various conditions for global characterization of change [24].

## 2. Materials and Methods

### 2.1. Growth of Bacterial Strains

Wild type^W^ *Clostridium perfringens* strains VPI, NCTR, ATCC 3626, and ATCC 13124 and their respective norfloxacin-resistant^NR^, ciprofloxacin-resistant^CR^, and gatifloxacin-resistant^GR^ mutants were used in this study (Table 1). All of the mutants generated* in vitro* using large concentrations of fluoroquinolones had stable mutations in gyrase A genes and some also had mutations in topoisomerase genes [25]. Brain heart infusion (BHI) broth (Remel, Lenexa, KS), with vitamin K (1 *μ*g/mL) and hemin (5 *μ*g/mL, Sigma Chemical Co., St. Louis, MO) but without antibiotics, was used for growth of the bacteria [25]. Cell preparation, inoculation, and incubation for all assays were performed in a glove box with an anaerobic atmosphere of 85% N_2_, 10% CO_2_, and 5% H_2_ at 37°C.

### 2.2. Phenotype Microarrays

A phenotypic microarray experiment was performed in 96-well microtiter plates, using PM 1–20 plates (Biolog, Inc., Hayward, CA) that contained different nutrients, chemicals, or inhibitory substances in each well, as described by Bochner [24]: PM 1-2, carbon source; PM 3, nitrogen source; PM 4, phosphorus and sulfur sources; PM 5, nutrient supplements; PM 6–8, peptides and nitrogen sources; PM 9, osmolytes; PM 10, pH values; PM 11–20, various chemicals, including antimicrobial agents. The manufacturer's instructions were followed and the assays were performed using their reagents. The wild types and mutants from BHI tubes (Table 1) were grown on blood agar plates. The bacterial colonies were suspended in Biolog broth. The Biolog turbidimeter was used to measure cell density and the cells were diluted to 40% transmittance. The cells then were further diluted, according to the Biolog instructions, for use in specific plates, and 100 *μ*L of diluted cells was used for inoculation of each well. The plates were incubated anaerobically for 24–48 h at 37°C. The effect of different conditions on cell growth was estimated by measuring the cell density (A_750_) in each well, using a spectrophotometer, and comparing the growth with wells containing nonsupplemented Biolog broth. Statistical analysis was performed by Student's* t*-test.

### 2.3. Comparison of the Effects of Dipeptides on Wild Types and Resistant Strains

Two dilutions of each of the dipeptides Gly-Met, Gly-Phe, and Gly-Leu (Sigma) were prepared in the Biolog proprietary concentration range used in the Biolog PM 6–8 plates, using the medium recommended by Biolog. 100 *μ*L of each dilution was added to duplicate wells of 96-well microtiter plates, along with 100 *μ*L of each of the cells, prepared according to the instructions of Biolog. The microtiter plates were incubated at 37°C for 24–48 h and the optical density (A_750_) was measured by spectrophotometer. Control wells contained media without dipeptides.

### 2.4. Sensitivity of Wild Types and Fluoroquinolone-Resistant Mutants to Antimicrobial Agents and Ethidium Bromide

Comparison of the antimicrobial susceptibilities of different strains was performed by the Etest (bioMérieux, Inc., Durham, NC) according to the Clinical and Laboratory Standards Institute (CLSI) guidelines and manufacturer's instructions. The minimum inhibitory concentration (MIC) was measured for each of the mutants and the wild type of each strain.

Sensitivity of strains to ethidium bromide was measured by the agar dilution method, according to CLSI guidelines, using BHI agar containing 0, 2, 4, 5, 6, 8, and 10 *μ*g/mL ethidium bromide. The plates were inoculated with 5 *μ*L of an overnight culture of each strain and were examined for growth following incubation.

### 2.5. Microarray Analysis

The microarray analysis of gatifloxacin-resistant mutants 13124^GR^ and NCTR^GR^ and their respective wild type was performed as previously described [21]. Briefly, the exponential growth phase of cultures of strains grown in BHI was used for RNA extraction for microarray analysis. RNA-Bee reagents from TEL_TEST, Inc. (Friendship, TX) and the RNeasy Mini Kit from QIAGEN, Inc. (Valencia, CA) were used to purify RNA according to the manufacturers' instructions. RNase free DNase 1 (Boehringer-Mannheim, Ingelheim, Germany) was used to remove contaminating DNA. The RNA was quantified using a Nanodrop ND-1000 spectrophotometer (NanoDropTechnology, Wilmington, DE). The probes for hybridization to RNA for microarray analysis were designed by Biodiscovery LLC (Ann Arbor, MI) (http://www.mycroarray.com). For the comparison of wild type and gatifloxacin-resistant mutants of NCTR and 13124, the known sequences of* C. perfringens* strains 13 and 13124, respectively, from the GenBank were used to design probes [21]. The designs of these probes can be accessed at the following websites: for NCTR at http://www.ebi.ac.uk/arrayexpress/arrays/A-MEXP-2027 and for 13124 at http://www.ebi.ac.uk/arrayexpress/arrays/A-MEXP-2008 [21].

The hybridization and analysis of RNA of each strain to the array probes were performed by Biodiscovery LLC, using Fluor-labeled RNA [21]. At the completion of hybridization, the arrays were scanned in an Axon 4000B scanner (Molecular Devices, Sunnyvale, CA) using GenePixPro software (v 6.1.0.4). The experiments were repeated with three sets of RNA for each strain. For comparing the expressions of the genes in the wild types and the mutant strains, the mean expression of each of the genes in the mutants was divided by the mean expression of the same gene in the wild type [21].

## 3. Results

### 3.1. Effect of Fluoroquinolone Resistance Selection on the Ability of Strains to Grow at Different pH Values and in Different Osmolytes

The growth of strains of* C. perfringens* in Biolog PM 1–20 plates reflected the effects of various substrates and conditions on the metabolic activities of wild type and resistant strains. The effect of fluoroquinolone resistance selection on pH tolerance, measured in Biolog PM 10 plates, showed that resistance to fluoroquinolones affected the pH ranges in which the strains could grow or survive. Gatifloxacin resistance selection reduced the pH range of growth by 0.5–2 units. The effect was more pronounced in the gatifloxacin-resistant mutant NCTR^GR^, which could only grow or survive up to pH 8, unlike the wild type NCTR^W^ and others, which grew at pH 10 (*P* < 0.05).

The growth on different concentrations of osmolytes in Biolog PM 9 showed differences in osmotic tolerance among the wild types. In general, resistance selection to different fluoroquinolones affected tolerance to NaCl, urea, sodium lactate, and sodium nitrite differently (Table 2). Tolerance to NaCl was reduced in the norfloxacin- and gatifloxacin-resistant mutants 13124^NR^ and 13124^GR^ but increased in the ciprofloxacin-resistant mutant VPI^CR^ (*P* < 0.05). Other strains were not substantially affected (Table 2). Although ciprofloxacin-resistant strains 13124^CR^ grew better than wild types in the same concentrations of urea and NCTR^CR^ grew better than its wild type on the same concentration of sodium lactate and sodium nitrite (*P* < 0.05), in general, fluoroquinolone resistance selection decreased the tolerance (*P* < 0.05). Microarray data showed that the nitrite transporter gene similar to* CPE 1442* was downregulated 4.65-fold in the mutant NCTR^GR^, which corresponded to the decreased tolerance of this mutant to sodium nitrite (see Supplementary Table S1 available online at http://dx.doi.org/10.1155/2014/456979).

### 3.2. Effect of Fluoroquinolone Resistance Selection on Growth on Nutrients

Biolog PM 1 and PM 2 plates containing various substrates were used to detect the effect of resistance development on the utilization of carbon sources. In general, compounds that supported the growth of all four wild types also supported the growth of the resistant mutants to different extents, with some exceptions. Trehalose and sucrose did not support the growth of norfloxacin-resistant 13124^NR^ and gatifloxacin-resistant 13124^GR^. Growth of 13124^GR^ also was reduced on maltose. Microarray data also showed that the* CPF_1785* and* CPF_0541* genes, for the transport of sucrose and trehalose, respectively, into the cells, were downregulated 15- and 31-fold in a gatifloxacin-resistant mutant, 13124^GR^, and putative maltose transporters were also downregulated to a lesser extent (Supplementary Tables S2 and S3). The wild type strains also differed in their ability to metabolize some carbon sources. The wild type and resistant mutants of 13124 could grow on sorbitol, glycerol, and D-fructose-6-phosphate, whereas other wild type strains did not. Interestingly, ciprofloxacin-resistant 3626^CR^ could grow on fructose and D-fructose 6-phosphate, but the wild type 3626^W^ and mutants 3626^NR^ and 3626^GR^ could not grow.

**Figure 1:**
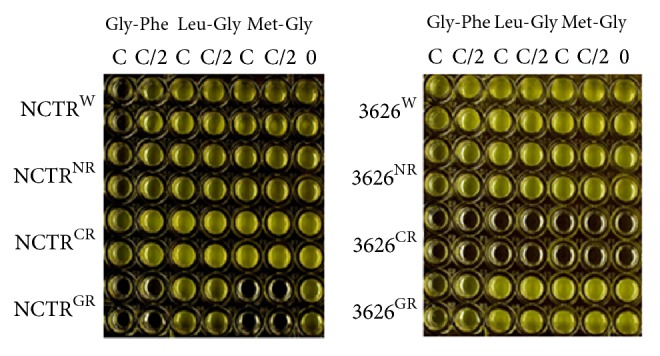
Comparison of growth of fluoroquinolone-resistant mutants of NCTR and 3626 with the growth of the wild types in the presence of three dipeptides. W, NR, CR, and GR refer to wild type, norfloxacin-resistant, ciprofloxacin-resistant, and gatifloxacin-resistant, respectively. Statistically significant differences were observed in the growth of strains (*P* < 0.05). C indicates the dipeptide concentration equivalent to the proprietary concentration of the compounds in the Biolog microtiter plate that had affected the bacterial growth or survival. C/2 is half of the concentration of dipeptide used in the Biolog microtiter plate. 0 is Biolog medium with no dipeptide added. This medium was used in all the wells for the dipeptide experiments. The dipeptides used in this experiment were selected based on the effects observed in experiments using Biolog plates.

The chemicals used as sources of nitrogen, phosphorus, and sulfur in the Biolog PM 3–8 plates that supported the growth of wild types also supported the fluoroquinolone-resistant mutants to different extents, with the following exceptions. Incubation of strains in the wells containing several dipeptides, including those dipeptides containing Gly, Leu, and Met, resulted in a decrease in the OD of some of the strains, compared with the negative control, in which no additional compound was present. This indicates cell lysis. Three of the dipeptides, Gly-Phe, Leu-Gly, and Met-Gly, were used in a separate experiment to confirm the inhibitory effect of dipeptides on some of the strains (Figure 1). Gly-Phe and Met-Gly inhibited the growth of gatifloxacin-resistant NCTR^GR^ at two different concentrations (*P* < 0.05). At the higher concentrations, Gly-Phe also inhibited the growth of the wild type NCTR^W^, NCTR^NR^, and 3626^GR^, but to a lesser extent (Figure 1). Wild type strain 3626^W^ and mutant strains 3626^NR^ and 3626^GR^ could grow in the control media without any dipeptide, but 3626^CR^ could not grow. The requirements for growth of this mutant had changed and were different from those of 3626^W^, 3628^NR^, and 3628^GR^, so it could not grow in the media that supported the others (Figure 1).

### 3.3. Effect of Fluoroquinolone Resistance Selection on the Tolerance of Strains to Antimicrobials and Ethidium Bromide

Fluoroquinolone resistance selection, in general, did not substantially alter the sensitivity of different* C. perfringens* strains to most compounds included in Biolog PM 11–20. Results of Etests, however, showed variation in the effect of fluoroquinolone resistance selection on the susceptibilities of strains to seven antimicrobial agents (Table 3). Gentamicin and erythromycin susceptibilities increased in all resistant strains except the norfloxacin-resistant VPI^NR^ and NCTR^NR^. Susceptibilities to other drugs also increased to different extents in some resistant strains in comparison to their respective wild types. Some resistant strains were less susceptible than their wild types to amoxicillin, cefoxitin, ceftriaxone, chloramphenicol, and metronidazole (Table 3). Strains 3626^NR^ and 3626^GR^ were less susceptible than their wild types to amoxicillin, chloramphenicol, and metronidazole and 3626^GR^, NCTR^NR^, NCTR^GR^, VPI^NR^, VPI^CR^, and VPI^GR^ were less susceptible than the wild types to cefoxitin (Table 3). VPI^GR^ was also less susceptible than its wild type to amoxicillin, ceftriaxone, and chloramphenicol.

Fluoroquinolone resistance selection also affected sensitivities of resistant strains to ethidium bromide (Table 3). Wild type* C. perfringens *NCTR^W^ was the most sensitive strain (MIC = 2 *μ*g/mL) to ethidium bromide, followed by 13124^W^ (MIC = 4 *μ*g/mL). The sensitivities of all resistant mutants of NCTR to ethidium bromide were substantially decreased, but sensitivities of norfloxacin- and gatifloxacin-resistant strains 13124^NR^ and 13124^GR^ to ethidium bromide increased. Norfloxacin and ciprofloxacin resistance selection also decreased the sensitivity of 3626 and VPI to ethidium bromide, but gatifloxacin-resistant 3626^GR^ became more sensitive to it.

## 4. Discussion

We have investigated the global changes in the metabolic activities associated with resistance development to three fluoroquinolones in four different strains of* C. perfringens.* Fluoroquinolone resistance selection affected the strains differently in their abilities to metabolize nutrients, grow at different pH values, and tolerate different osmolytes, antimicrobial agents, and other chemicals.

Ciprofloxacin and gatifloxacin resistance selection had opposite effects on the carbohydrate metabolism of strains 3626^CR^ and 13124^GR^. Unlike the wild type, 3626^CR^ grew on fructose and fructose 6-phosphate. Strain 13124^GR^ could not grow on sucrose and trehalose and it showed reduced growth on maltose. Downregulation of genes involved in the sucrose and trehalose-specific phosphotransferase (PTS) systems* CPF_1785* and* CPF_0541*, the maltose ABC transporter* CPF_2652*, and a putative maltose transporter* CPF_2654* in the gatifloxacin-resistant strain 13124^GR^ was observed, which may have resulted in the lack of growth of 13124^GR^ on sucrose and trehalose and the decreased growth on maltose (Supplementary Tables S2-S3).

Fluoroquinolone resistance selection also reduced the ability of NCTR^GR^ to grow in alkaline pH; it decreased the osmotic tolerance of gatifloxacin-resistant strains 13124^GR^ more than others (Table 2).

Bacterial adaptation response to alkaline pH and tolerance to hyperosmolarity could be related to alteration in the expression of transporter genes, in membrane permeability, or in osmoprotective substances [26, 27]. Lack of growth of strain NCTR^GR^ in alkaline pH could result from more than threefold downregulation of the membrane-spanning transporter protein similar to* CPE0166* of* C. perfringens* strain 13 in this strain. Likewise, the membrane lipoprotein TmPC precursor gene similar to* CPE1580* of* C. perfringens* strain 13 was downregulated 4.95- fold and the nitrite transporter gene similar to* CPE1442* of* C. perfringens* strain 13 was downregulated 4.65-fold in strain NCTR^GR^, which may have reduced the osmotolerance of this strain (Supplementary Table S1). Furthermore, several transporters and ten other putative membrane proteins were downregulated at least 1.5 times in strain 13124^GR^  (Supplementary Tables S2 and S3). Further investigation will be needed to elucidate the roles of these genes.

The metabolism of dipeptides was also affected differently in the resistant strains (Figure 1). The lack of growth of NCTR^GR^ with Gly-Phe and Met-Gly (Figure 1) was not related to nutritional deficiency or pH change, since NCTR^GR^ could grow in media not supplemented with these chemicals, whose addition did not change the pH. Some cyclic dipeptides have been shown to make cell membranes permeable, resulting in cell lysis, with effects that are strain-specific [28, 29]. The inhibitory effect of dipeptides could result from alteration in the transport mechanism, in membrane structure, or in dipeptidase production. Microarray results showed downregulation of a gene similar to* CPE1928* of* C. perfringens* strain 13, a probable dipeptidase gene, in NCTR^GR^. Gatifloxacin resistance selection also affected survival of 3626^GR^ with Gly-Phe. Ciprofloxacin-resistant mutant 3626^CR^, which was the only strain that grew on fructose and fructose 6-phosphate, could not grow on the medium used to assay the effect of dipeptides either with or without these compounds, indicating that it had different growth requirements from the others.

The antimicrobial susceptibility assay showed that wild type strains differed in their level of susceptibility to different antimicrobial agents, and fluoroquinolone resistance affected the strains differently. Strain 13124^W^, which is a clinical gangrene isolate [2], was more resistant than others to some antimicrobial agents, especially to ceftriaxone and gentamicin. In most cases, the susceptibility of fluoroquinolone-resistant strains to other antimicrobial agents decreased, but a 2–4-fold increase in resistance was also observed, including *β*-lactams. Microarray data indicated that the expression of some *β*-lactamase genes in the resistant strains was downregulated, although upregulation of other *β*-lactamase genes was observed. Previously it has been shown in* Salmonella enterica* that susceptibilities to unrelated antibiotics are influenced by mutations in gyrase genes [9]. Also, changes in cellular permeability, decreases in efflux of antimicrobial agents because of changes in membrane proteins, and downregulation of some transporters as shown in microarrays could have contributed to alterations in susceptibility (Supplementary Tables S1–S3). Our results reflect the epidemiological studies that have conflicting accounts of the relationships between use of fluoroquinolones and isolation of bacteria that are either more susceptible to or resistant to other antimicrobial agents [12, 13, 15, 16, 18].

Substantial and opposite effects of resistance selection to norfloxacin and gatifloxacin on the ability of 13124 and NCTR to grow on ethidium bromide were observed. Microarray results showed that the expression of a multidrug-efflux transporter gene similar to* CPE1604* of* C. perfringens* strain 13 was upregulated 11.25 times in NCTR^GR^, which could have contributed to the efflux of ethidium bromide, resulting in tolerance to higher concentrations (Supplementary Table S1). Considering the decrease in the antibiotic susceptibilities of strains to other drugs, most likely this gene was not involved in their efflux. We previously showed that a transport gene similar to* CPE1506* of* C. perfringens* strain 13 cloned into strain VPI also contributed to the efflux of ethidium bromide in the recombinant VPI strain [30].

In conclusion, fluoroquinolone resistance selection resulted in changes in various metabolic activities in different strains of* C. perfringens.* These changes were influenced by both the structures of the bacterial genomes and the drugs that were used. It has been shown that both the structure of the fluoroquinolone and the bacterial genotype affect the colonization efficiency of* C. difficile* strains in hamsters [10]. Strain-specific effects may explain some of the apparently conflicting reports on the effects of clinical use of fluoroquinolones on virulence and antimicrobial susceptibility in other species of bacteria [12, 13, 15–18]. The interaction of different fluoroquinolones with* C. perfringens* and other pathogenic bacteria merits further investigation.

## Supplementary Material

Alteration of the expression of various genes in the gatifloxacin resistant strains of Clostridium perfringens as detected by microarray analysis.

## Figures and Tables

**Table 1:** Wild types and fluoroquinolone-resistant mutants of *C*. *perfringens* used in this study with stable mutations in *gyrA* and *parC *resulting in amino acid conversion.

*C*. *perfringens* strain	Wild type	Norfloxacin-resistant^NR^	Ciprofloxacin-resistant^CR^	Gatifloxacin-resistant^GR^
VPI	—	D87Y *gyrA*, V196F *parC *	D87Y *gyrA*, D87Y *parC *	G81C *gyrA*, D93Y and D502Y *parC *
NCTR	—	D87Y *gyrA *	D87Y *gyrA *	G81C and D87Y *gyrA *
3626	—	G81C *gyrA*, D87Y *parC *	D87Y *gyrA*, D93Y *parC *	G81C and D87Y *gyrA*, D93Y and A131S *parC *
13124	—	A119E	D87Y *gyrA*, S89I *gyrA *	G81C and D93Y *gyrA*, S89I *parC *

**Table 2:** Effect of fluoroquinolone resistance selection on the growth of *C*. *perfringens* strains (shown by OD_750_) with different concentrations of sodium chloride, urea, sodium lactate, and sodium nitrite^a^.

*C*. *perfringens* strain	Compound	Wild type	Norfloxacin-resistant	Ciprofloxacin-resistant	Gatifloxacin-resistant
VPI	Sodium chloride	1%	1%	4%^∗b^	2%
NCTR		2%	2%	2%	1%
3626		2%	2%	2%	2%
13124		6.5%	4%^*^	6%	2%^*^

VPI	Urea	6%	6%	4%	4%^*^
NCTR		6%	4%	3%	2%^*^
3626		3%	4%	4%	2%
13124		7%	6%	7%^∗∗c^	6%^*^

VPI	Sodium lactate	1%	1%	2%^*^	2%
NCTR		2%	1%	2%^**^	2%
3626		1%	2%	2%	2%
13124		6%	4%^*^	5%^*^	3%^**^

VPI	Sodium nitrite	60 mM	40 mM^*^	40 mM^*^	60 mM
NCTR		60 mM	40 mM	60 mM^**^	20 mM^*^
3626		20 mM	40 mM	40 mM	20 mM
13124		60 mM	60 mM	60 mM	40 mM^*^

^a^The percent (%) or mM value indicates the concentration in which the strain would grow. ^b∗^Statistically significant differences are marked by asterisks (*P* < 0.05). ^c∗∗^Better growth was observed for the mutant than for the wild type (*P* < 0.05) at the concentrations marked by double asterisks.

**Table 3:** Comparison of the effect of fluoroquinolone resistance selection on the MIC of various antimicrobial agents and ethidium bromide. Ethidium bromide concentrations in the plates were 0, 2, 4, 5, 6, 8, and 10 *µ*g/mL.

*C. perfringens* strains	MIC (*µ*g/mL), as shown by Etest for antimicrobial agents or agar dilution for ethidium bromide							
	Erythromycin	Amoxicillin	Ceftriaxone	Gentamicin	Chloramphenicol	Cefoxitin	Metronidazole	Ethidium bromide
3626								
W	2	0.1	1	128	3	1	1.5	6
NR	1.5	0.19^∗a^	0.016	48	8^*^	0.25	3^*^	10^∗∗b^
CR	0.5	0.1	4^*^	12	2	1	1.5	10^**^
GR	1	0.25^*^	0.016	64	6^*^	2^*^	3^*^	5
13124								
W	2	0.25	64	512	8	4	3	4
NR	0.75	0.125	16	64	3	1.5	1.5	2
CR	1.5	0.75^*^	32	128	8	2	4^*^	4
GR	1	2^*^	12	256	3	4	1.5	2
NCTR								
W	2	0.13	4	128	4	0.38	1.5	2
NR	0.75	0.2	0.016	128	3	0.75^*^	0.75	10^**^
CR	1	0.2	3	48	4	0.5	1	10^**^
GR	0.75	0.1	1	32	3	1^*^	1.5	10^**^
VPI								
W	1.5	0.25	16	384	3	0.75	3	6
NR	3	0.38	16	64	4	1.5^*^	3	8^**^
CR	1.5	1.5^*^	16	256	3	3^*^	2	10^**^
GR	0.75	0.5^*^	64^*^	256	6^*^	2^*^	3	8^**^

^a^The ∗ indicates that fluoroquinolone resistance selection resulted in a decrease in susceptibility; W, NR, CR, and GR refer to wild type, norfloxacin-resistant, ciprofloxacin-resistant, and gatifloxacin-resistant, respectively. ^b∗∗^Resistant strains grew on the plates containing 10 *µ*g/mL of ethidium bromide, so the MIC of the ethidium bromide was greater than 10 *µ*g/mL for these strains.
