# Supplementary material for: Global Phenotypic Characterization of Effects of Fluoroquinolone Resistance Selection on the Metabolic Activities and Drug Susceptibilities of Clostridium perfringens Strains
Source: Int J Microbiol. 2014 Dec 21;2014:456979. doi: 10.1155/2014/456979 (PMC4283427; doi:10.1155/2014/456979)
Supplement: Supplementary file 1 — Alteration of the expression of various genes in the gatifloxacin resistant strains of Clostridium perfringens as detected by microarray analysis. [file 456979.f1.docx]

Table S1: Alteration of level of expression of various transporter and membrane proteins in gatifloxacin-resistant *C. perfringens* NCTR^GR^. The sequence of *C. perfringens* strain 13 was used to design probes for microarray analysis of *C. perfringens* strain NCTR, so the altered genes reported in the table are similar to CPE genes of *C. perfringens* strain 13.

| Gene | Description | Fold change  mutant/wild type |
| --- | --- | --- |
| *CPE0584* | PTS system | -8.93 |
| *CPE2228* | ethanolamine transporter | -5.18 ^a^ |
| *CPE1580* | membrane lipoprotein TmpC precursor | -4.95 |
| *CPE1442* | nitrite transporter | -4.65 |
| *CPE0166* | membrane-spanning transporter protein | -3.38 |
| *CPE0821* | PTS system | -1.81 |
| *CPE0061* | integral membrane transport protein | -1.53 |
| *CPE0404* | branched-chain amino acid transporter | 1.47 |
| *CPE2084* | molybdenum ABC transporter | 1.81 |
| *CPE1969* | spermidine/putrescine ABC transporter | 2.64 |
| *CPE1970* | spermidine/putrescine ABC transporter | 3.42 |
| *CPE1167* | glutamate/ aspartate transporter | 3.70 |
| *CPE1505* | uracil transporter | 3.78 |
| *CPE1465* | PTS system | 4.08 |
| *CPE0397* | transporter | 6.05 |
| *CPE0337* | amino acid transporter | 6.36 |
| *CPE1463* | PTS system | 7.64 |
| *CPE0963* | transporter | 10.65 |
| *CPE1604* | multidrug-efflux transporter | 11.25 |
| *CPE1466* | PTS system | 16.53 |

^a^ Growth of strain NCTR^GR^ on ethanolamine in the Biolog plate also was less than that of the wild type.

Table S2: Fold changes in gene expression found by microarray analysis in two gatifloxacin-resistant strains of *C. perfringens*, 13124^GR^ and NCTR^GR^, compared with the wild types. Negative values indicate the fold downregulation of a gene in the resistant strain compared with the wild type. The sequence of *C. perfringens* strain 13 was used to design probes for microarray analysis of *C. perfringens* strain NCTR, so the altered genes reported in the table are similar to CPE genes of *C. perfringens* strain 13.

| Description | Genes from  *C. perfringens* strains | | Fold change  (mutant/wild) | |
| --- | --- | --- | --- | --- |
|  | 13124 | NCTR | 13124 | NCTR |
| PTS system (trehalose-specific IIBC component) | *CPF_0541* | *CPE0561* | -31.53 | -1.42 |
| amino acid transporter | *CPF_0581* | *CPE0600* | -3.41 | -3.97 |
| ABC transporter | *CPF_0703* | *CPE0707* | -3.25 | 1.45 |
| ABC transporter | *CPF_1426* | *CPE1218* | 2.01 | -1.53 |
| magnesium transporter | *CPF_1450* | *CPE1240* | -1.66 | 8.64 |
| ABC transporter | *CPF_1710* | *CPE1457* | 1.56 | 1.06 |
| ABC transporter | *CPF_1721* | *CPE1470* | -1.96 | 1.25 |
| ABC transporter | *CPF_1757* | *CPE1506* | -1.71 | -1.31 |
| PTS system (sucrose-specific IIBC component) | *CPF_1785* | *CPE1534* | -15.33 | -2.50 |
| ABC transporter | *CPF_1791* | *CPE1540* | -2.35 | -1.10 |
| spermidine/putrescine ABC transporter | *CPF_2226* | *CPE1971* | 1.53 | 3.12 |
| amino acid ABC transporter | *CPF_2350* | *CPE2093* | -2.12 | 1.39 |
| maltose ABC transporter | *CPF_2652* | *CPE2343* | -2.85 | 2.93 |
| nucleoside transporter | *CPF_2819* | *CPE2496* | 2.23 | -1.12 |
| putative inner membrane protein translocase | *CPF_2994* | *CPE2657* | -1.53 | -1.33 |

Table S3: Alteration of the levels of expression of various transporters and membrane proteins

in gatifloxacin-resistant mutant *C. perfringens* 13124^GR^

| Gene | Description | Fold change  mutant/wild type |
| --- | --- | --- |
| *CPF_0070* | putative PTS system | -13.89 |
| *CPF_0071* | PTS system, N-acetylglucosamine-specific IIBC | -5.26 |
| *CPF_1883* | ribose ABC transporter, ribose-binding protein | -5.20 |
| *CPF_0155* | putative membrane protein | -4.05 |
| *CPF_1882* | ribose ABC transporter, ATP-binding protein | -3.37 |
| *CPF_1831* | ABC transporter, ATP-binding protein | -2.81 |
| *CPF_1881* | ribose ABC transporter, permease protein | -2.54 |
| *CPF_2349* | amino acid ABC transporter, ATP-binding protein | -2.51 |
| *CPF_2341* | molybdate ABC transporter, permease protein | -2.50 |
| *CPF_1550* | galactoside ABC transporter, permease protein | -2.37 |
| *CPF_0256* | putative membrane protein | -2.29 |
| *CPF_1549* | galactoside ABC transporter, ATP-binding | -2.23 |
| *CPF_0538* | glycine betaine/L-proline ABC transporter | -2.23 |
| *CPF_1622* | probable proton-coupled thiamine transporter | -2.22 |
| *CPF_0704* | putative ABC transporter, permease protein | -2.21 |
| *CPF_1345* | putative membrane protein | -2.13 |
| *CPF_0174* | putative ABC transporter, permease protein | -2.11 |
| *CPF_2425* | putative membrane protein | -2.09 |
| *CPF_0102* | putative membrane protein | -1.98 |
| *CPF_0175* | ABC transporter, ATP-binding protein | -1.86 |
| *CPF_1846* | putative membrane protein | -1.78 |
| *CPF_1548* | putative galactoside ABC transporter | -1.76 |
| *CPF_2654* | putative maltose/maltodextrin ABC transporter | -1.75 |
| *CPF_0114* | putative membrane protein | -1.73 |
| *CPF_1457* | putative membrane protein | -1.72 |
| *CPF_0507* | ABC transporter, permease/ATP-binding protein | -1.71 |
| *CPF_0716* | putative membrane protein | -1.61 |
| Table S3 continued |  |  |
|  |  |  |
| *CPF_2263* | putative membrane protein | -1.60 |
| *CPF_2169* | putative membrane protein | -1.60 |
| *CPF_1816* | membrane protein, DedA family | -1.59 |
| *CPF_0183* | putative membrane protein | -1.59 |
| *CPF_1242* | putative dicarboxylate transporter | -1.57 |
| *CPF_0036* | putative transporter | -1.52 |
| *CPF_0129* | putative membrane protein | 1.53 |
| *CPF_0820* | PTS system, mannose/fructose/sorbose family, IID | 1.73 |
| *CPF_2413* | ABC transporter, substrate-binding protein | 1.78 |
| *CPF_1749* | putative membrane protein | 1.81 |
| *CPF_1298* | iron chelate uptake ABC transporter, FeCT | 2.00 |
| *CPF_0142* | putative membrane protein | 2.04 |
| *CPF_0787* | putative membrane protein | 2.05 |
| *CPF­ _1372* | putative membrane protein | 2.05 |
| *CPF_2336* | putative membrane protein | 2.10 |
| *CPF_2259* | putative membrane protein | 2.15 |
| *CPF_0516* | putative membrane protein | 2.29 |
| *CPF_0188* | cobalt ABC transporter, ATP-binding protein | 2.39 |
| *CPF_1235* | potassium transporter | 2.92 |
| *CPF_2316* | putative membrane protein | 3.12 |
